# Supplementary material for: Frequency distribution of journalistic attention for scientific studies and scientific sources: An input–output analysis
Source: PLoS One. 2020 Nov 11;15(11):e0241376. doi: 10.1371/journal.pone.0241376 (PMC7657513; doi:10.1371/journal.pone.0241376)
Supplement: Attachment — Submitted filename: Response to Reviewers.docx [file pone.0241376.s002.docx]

| KIT \| Institute of Technology Futures \| Englerstr. 2 \| Karlsruhe, Germany | **Institute of Technology Futures**  **Department of Science Communication**  Prof. Dr. Markus Lehmkuhl  Englerstr. 2, Building 20.30  76131 Karlsruhe, Germany  Phone: +49 721-608-41631  Email: markus.lehmkuhl@kit.edu  Web: <http://wmk.itz.kit.edu/3163.php>  Date: 2020-07-09 | |
| --- | --- | --- |
| PLOS ONE Editorial Board |  |  |
|  |  |  |
|  |  |  |

**Response to reviewers regarding** **our manuscript submitted for PLOS ONE**

Dear Editor,

Thank you for the constructive review of our manuscript titled “Frequency distribution of journalistic attention for scientific studies and scientific sources: An input – output analysis”, you have made very helpful remarks and rightly pointed out some aspects that still needed work. We have revised our manuscript according to your recommendations and feel that this has improved the quality of our work. In the following, we address each of the points mentioned in the review.

- *You indicated that you followed methods given in ref. 84: "Kube L. Konsensanalyse der Berichterstattung in den Wissenschaftsressorts deutscher Tageszeitungen. Unveröffentlichte Bachelorarbeit. 2018." […]*
  - This was a reference error in the manuscript, we did not use methods from ref. 84, but from ref. 86 (“Clauset A, Shalizi CR, Newman MEJ. Power-Law Distributions in Empirical Data. SIAM Rev. 2009; 51: 661–703. doi: 10.1137/070710111.”) and we described these methods in detail in the Materials and methods part of our study. We have corrected the reference.
- *Table 1 includes the New Scientist as one of the sources for published research. However the New Scientist does not publish original research. I believe a note should be added to table caption letting the readers know that the authors are aware of this point.*
  - We have added a footnote to Table 1 with the following content: “While the New Scientist was identified as one of the most important external sources for the journalistic coverage of biomedical research in [67], it differs from the other sources listed in the table in that it does not publish original research. This also explains why the New Scientist cannot be found in the results of our analyses.”
- *The authors repeatedly write that "y decreases exponentially with x" (lines 224, 291, 303, 308, 320, 491, 503). This statement is incorrect. The correct statement is "log y decreases linearly with log x" or "y decreases exponentially with log x".*
  - We have corrected all mentioned statements, writing that y decreases exponentially with the logarithm of x or that y decreases by 1/x^α^.
- *Since the hypothesis tested in the study involve power-laws, all graphs should be double-logarithmic. This applies already to Figs 1 and 2. For figure 2a, I recommend having the squares filled white since it will make it easier to distinguish the symbols in the figure.*
  - We have changed Figure 1 and Figure 2 to logarithmic axes and changed the fillings of the symbols in Figure 2a. Additionally, we noticed that the references in the labels were wrong and have corrected them.
- *I am not sure that the evidence from Fig. 1 is enough to propose that the exponent should be expected to be close to 1 since the data available is not a good sample of the entire population. This caveat is actually the reason why this study is important*.
  - We have removed the value of the exponent from hypothesis 1.
- *Concerning the power law exponent, its value is related to the concentration of impact. With regard to income and wealth, one number that quantifies their concentration in the hands of a few people is the Gini coefficient. A power law distribution with an exponent alpha > 1 results in a Gini coefficient of 1/(2*alpha - 1).*
  - We think that a discussion of the relationship between the Gini coefficient and the power law exponent would exceed the scope of our study. But we thank the editor for this comment, we are already working on another study in which we focus on the Gini coefficient of the distributions of source selection and selection of single results in science journalism. In this study, we will calculate the Gini coefficient independent of a power law fit.
- *In the discussion of the results in Figs 1 and 2, I would remove the R^2 values. I do not think the data is of good enough quality to warrant the quantification involved in the R^2 calculation.*
  - We have removed the R^2 values.
- *In the Materials and Methods, the authors refer to the sources of the input and output data. I believe that Scopus is a very good source for publications. I think that Altmetric is not as good a source. The reason is that while coverage in Scopus has likely remained pretty constant during the study period, I doubt that the same is true for Altmetric. That means that results for 2014 are not comparable to results for 2018.
  In fact, I strongly disagree with the statement in lines 333-335. The lack of coverage by Altmetric should left censor a large fraction of SIPs published in the earlier years of the studied period.
  I suggest that the authors add a new figure showing (a) how the number of SIPs changes by time period, (b) how the number of distinct scientific journals publishing SIPs changes by time period, and (c) how the number of distinct news sources covers by Altmetric changes by time period*.
  - We have changed the paragraph mentioned above (lines 333-335) and changed its position in the text. We have added a figure showing a) and b) (Fig 3a,b), but we are not able to show c) because Altmetric does not publish information concerning the development of the source base they use to calculate the MSM score. Additionally, we discuss the implications of the changes in the number of SIPs and journals with SIPs for our results in the results and discussion part and only use the last two years for the aggregated fits.
- *I recommend removing Figs. 3 and 4 and replacing them with a 2 panel figure in which the first panel shows the data (use lines of different colors instead of circles) and a fit line for just the last time periods. The second panel shows the aggregated data for the years for which Altmetric sampling is appropriate (probably the last two years of the data).
  I recommend that the same be done with Figs. 5 and 6*
  - We have created two new figures (Fig 4a, b and 5a, b) showing only the data and fits for the last two years.
- *I think that the discussion of model selection should come before showing quality of fits for the power law model and the values of the fit parameters.*
  - We feel that changing the order of these paragraphs would not correspond to the logic of our analysis. Our overall goal was to test the hypotheses that a power law is a plausible model for the journalistic selection of scientific sources and of single study results. Our focus was mostly on the power law distribution. Therefore, our first steps were to estimate the power law fits and check their goodness-of-fit. Only after these steps, we could compare our model to other candidate distributions. While we understand the editor’s recommendation, we would like to leave the order of these paragraphs as it is. We have added an additional paragraph in the discussion in which we stress that other distributions are better fits.
- *Table 3 should appear before Table 2 and the Table should also include a comparison between the Weibull and the truncated power law.
  Table 2 should be separate into two tables. And fit parameters should be given for both power law and truncated power law. By the way, the truncated poor law model seems to be the better one for both data sets.*
  - We have changed the order of tables and added a column for the missing comparison. We have separated table 2 into two tables and added columns for the alpha- and lambda-parameter of the truncated power law.
- *However, it is not clear that the data retrieved from Altmetric for news sources is being shared (line 363). If it is not, it should be.*
  - We do share a dataset containing all SIPs with their title, authors, the journals they were published in, the DOI, the year of publication and the MSM score (S1 File). We are not able to share data about the individual news sources that mentioned the studies because we did not have access to this information. We only retrieved the value of the MSM score.
- *It would be interesting to provide an analysis of the distribution of the topics of the SIPs. I realize that classifying nearly 6000 papers by hand is tricky. However, the authors could consider using the topic classification algorithm by Lancichinetti et al (Physical Review X 2015) on the titles and abstracts of the SIPs.
  Or that analysis could be left for another paper...*
  - We think that an analysis of the distribution of the topics of the Social Impact Papers exceeds the scope of this paper, in which our main goal is to determine the structure of the journalistic selection of research results. But we agree that it will be interesting to conduct such an analysis and would like to thank the editor for recommending an algorithm that could be used to classify the texts automatically.
- *In line 79, the authors write that "journalists can no longer unconditionally trust...". I am not sure the situation has really changed. what has maybe changes is the awareness of what is the potential for hyperbole or outright lies by scientists.*
  - We think that it is not important whether the situation or journalists’ awareness of the situation has changed, because the result would be the same. That being said, we would argue that both the situation and the awareness for the situation have changed.
- *The sentence in line 171 "thus the bond between ..." is not at all clear to me.*
  - We have changed the sentence to “Such numbers illustrate the extent to which the bond between journalism and a sufficiently large audience is eroding.”
- *In line 185, I think it should be "value" instead of "values"; Line 350, "for" instead of "from"*
  - We have changed both words.
- *Line 414: Writing "fewer than 1 in 10,000" is more informative than "about 0.07 percent"*
  - We have changed the wording, but it is “fewer than 1 in 1,000”, not “fewer than 1 in 10,000”.

Additionally, we have made some minor changes concerning the wording and qualified some of our statements in the discussion.

We hope that we were able to address all of your concerns and that our manuscript now meets PLOS ONE’s publication criteria. We are looking forward to receiving a favorable response from you regarding the acceptance of our manuscript.

Yours sincerely,

Prof. Dr. Markus Lehmkuhl
