# Supplementary material for: Frequency distribution of journalistic attention for scientific studies and scientific sources: An input–output analysis
Source: PLoS One. 2020 Nov 11;15(11):e0241376. doi: 10.1371/journal.pone.0241376 (PMC7657513; doi:10.1371/journal.pone.0241376)
Supplement: Attachment — Submitted filename: Response to Reviewers.docx [file pone.0241376.s003.docx]

| KIT \| Institute of Technology Futures \| Englerstr. 2 \| Karlsruhe, Germany | **Institute of Technology Futures**  **Department of Science Communication**  Prof. Dr. Markus Lehmkuhl  Englerstr. 2, Building 20.30  76131 Karlsruhe, Germany  Phone: +49 721-608-41631  Email: markus.lehmkuhl@kit.edu  Web: <http://wmk.itz.kit.edu/3163.php>  Date: 2020-08-17 | |
| --- | --- | --- |
| PLOS ONE Editorial Board |  |  |
|  |  |  |
|  |  |  |

**Response to reviewers regarding** **our manuscript submitted for PLOS ONE**

Dear Editor,

Thank you for the second review of our manuscript titled “Frequency distribution of journalistic attention for scientific studies and scientific sources: An input – output analysis”. We are glad that our revisions were able to address your concerns and we have changed the remaining stylistic issues you mentioned:

- *Please always include the leading zero in numerical values '0.0001' instead of '.0001' (see, example, Tables 2-4). When presenting estimates for coefficients, do not include non-significant digits. For example, '2.4868 (0.1579)' should be replaced with '2.48 (0.16)' or '2.5 (0.2)'*
  - We have added leading zeros where missing and removed non-significant digits in all tables and in the text.
- *In Tables, you list values of the estimate and standard deviation. Is it really the standard deviation (SD) or is it the standard error (SE)? The SE of the estimate of a coefficient is more useful than the SD.*
  - The values listed in the tables are the standard deviation of the parameters in the bootstrap samples, i.e. the bootstrapped standard error. We have changed SD to SE and added a short explanation under tables 3 and 4 (“Standard errors of the parameters were estimated by bootstrapping.”).

Yours sincerely,

Prof. Dr. Markus Lehmkuhl
