# Supplementary material for: Frequency distribution of journalistic attention for scientific studies and scientific sources: An input–output analysis
Source: PLoS One. 2020 Nov 11;15(11):e0241376. doi: 10.1371/journal.pone.0241376 (PMC7657513; doi:10.1371/journal.pone.0241376)
Supplement: Attachment — Submitted filename: Response to Reviewers.docx [file pone.0241376.s004.docx]

| KIT \| Institute of Technology Futures \| Englerstr. 2 \| Karlsruhe, Germany | **Institute of Technology Futures**  **Department of Science Communication**  Prof. Dr. Markus Lehmkuhl  Englerstr. 2, Building 20.30  76131 Karlsruhe, Germany  Phone: +49 721-608-41631  Email: markus.lehmkuhl@kit.edu  Web: <http://wmk.itz.kit.edu/3163.php>  Date: 2020-09-09 | |
| --- | --- | --- |
| PLOS ONE Editorial Board |  |  |
|  |  |  |
|  |  |  |

**Response to reviewers regarding** **our manuscript submitted for PLOS ONE**

Dear Editor,

we have again checked each decimal number and rounded all of them to two decimals or at least two significant digits. We hope that they are correct now. The example of an incorrect rounding you gave in your review (0.9898 to 0.9 on page 22) is probably based on a misreading caused by the tracked changes mode. The number we rounded to 0.9 was 0.898 and not 0.9898. We have now changed it to 0.90. The version of the manuscript with tracked changes we uploaded shows the changes from the unrounded numbers as they were before the revision you criticized.

Yours sincerely,

Prof. Dr. Markus Lehmkuhl
